# Exploring causal networks underlying fat deposition and muscularity in pigs through the integration of phenotypic, genotypic and transcriptomic data

**DOI:** 10.1186/s12918-015-0207-6

**Published:** 2015-09-16

**Authors:** Francisco Peñagaricano, Bruno D. Valente, Juan P. Steibel, Ronald O. Bates, Catherine W. Ernst, Hasan Khatib, Guilherme JM Rosa

**Affiliations:** Department of Animal Sciences, University of Wisconsin-Madison, Madison, WI 53706 USA; Dairy Science, University of Wisconsin-Madison, Madison, WI 53706 USA; Department of Animal Science, Michigan State University, East Lansing, MI 48824 USA; Biostatistics and Medical Informatics, University of Wisconsin-Madison, Madison, WI 53706 USA; Present Address: Department of Animal Sciences, and University of Florida Genetics Institute, University of Florida, Gainesville, FL 326111 USA

**Keywords:** Causal inference, Complex traits, Networks, Pig meat quality

## Abstract

**Background:**

Joint modeling and analysis of phenotypic, genotypic and transcriptomic data have the potential to uncover the genetic control of gene activity and phenotypic variation, as well as shed light on the manner and extent of connectedness among these variables. Current studies mainly report associations, i.e. undirected connections among variables without causal interpretation. Knowledge regarding causal relationships among genes and phenotypes can be used to predict the behavior of complex systems, as well as to optimize management practices and selection strategies. Here, we performed a multistep procedure for inferring causal networks underlying carcass fat deposition and muscularity in pigs using multi-omics data obtained from an F_2_ Duroc x Pietrain resource pig population.

**Results:**

We initially explored marginal associations between genotypes and phenotypic and expression traits through whole-genome scans, and then, in genomic regions with multiple significant hits, we assessed gene-phenotype network reconstruction using causal structural learning algorithms. One genomic region on SSC6 showed significant associations with three relevant phenotypes, off-midline10th-rib backfat thickness, loin muscle weight, and average intramuscular fat percentage, and also with the expression of seven genes, including *ZNF24*, *SSX2IP*, and *AKR7A2*. The inferred network indicated that the genotype affects the three phenotypes mainly through the expression of several genes. Among the phenotypes, fat deposition traits negatively affected loin muscle weight.

**Conclusions:**

Our findings shed light on the antagonist relationship between carcass fat deposition and lean meat content in pigs. In addition, the procedure described in this study has the potential to unravel gene-phenotype networks underlying complex phenotypes.

## Background

Genetic linkage and association studies have been successful in identifying genomic regions associated with phenotypic traits in livestock species. Indeed, many quantitative trait loci (QTL) influencing different phenotypes have been reported in the last two decades [1]. However, the identification of the individual genes responsible for the phenotypic variation remains challenging. In addition, classical QTL mapping and association analysis do not provide in general any information about the molecular pathways involving the phenotype under study.

One way to unravel the molecular mechanisms underlying a phenotype of interest is to expand the type of traits under genetic analysis. One of such traits may be the abundance of messenger RNA transcripts, i.e., gene expression measurements. The combination of transcriptional profiling with genotypic information allows the mapping of genetic loci that control gene expression, commonly termed as expression quantitative trait loci [2, 3]. The co-localization of expression QTL (eQTL) with phenotypic QTL (pQTL) is commonly used to nominate candidate genes and identify causative variants. Indeed, the integration of phenotypic data with genotypic information and transcriptional profiling has the potential to uncover gene networks and the genetic control of gene activity, as well as shed light on the genetic architecture underlying phenotypic variation [4, 5].

Although genetical genomic studies can be used to provide evidence on the manner and extent of connectedness among phenotypic and expression traits, most often these connections have been explored only in terms of associations, i.e., connecting variables without causal direction. Indeed, a major goal in the study of complex traits is to uncover the causal relationships among the variables under study. In this context, the notion of *d*-separation and different causal inference methods [6] can be used to explore the universe of causal hypotheses in order to find a causal structure that is able to generate the observed pattern of conditional independencies between variables. Different approaches have been proposed for inferring causal relations in genetical genomics studies, including likelihood-based model selection [7], directed versions of the PC algorithm [8], structural equation models [9, 10], homogeneous conditional Gaussian regression models [11], and mixed graphical Markov models [12]. Causal claims about the relationship between QTL and phenotypic and expression traits are justified by the Mendelian randomization of alleles that occurs during meiosis and the unidirectional effect of genotype on both gene expression and phenotype [13, 14].

**Fig. 1:**
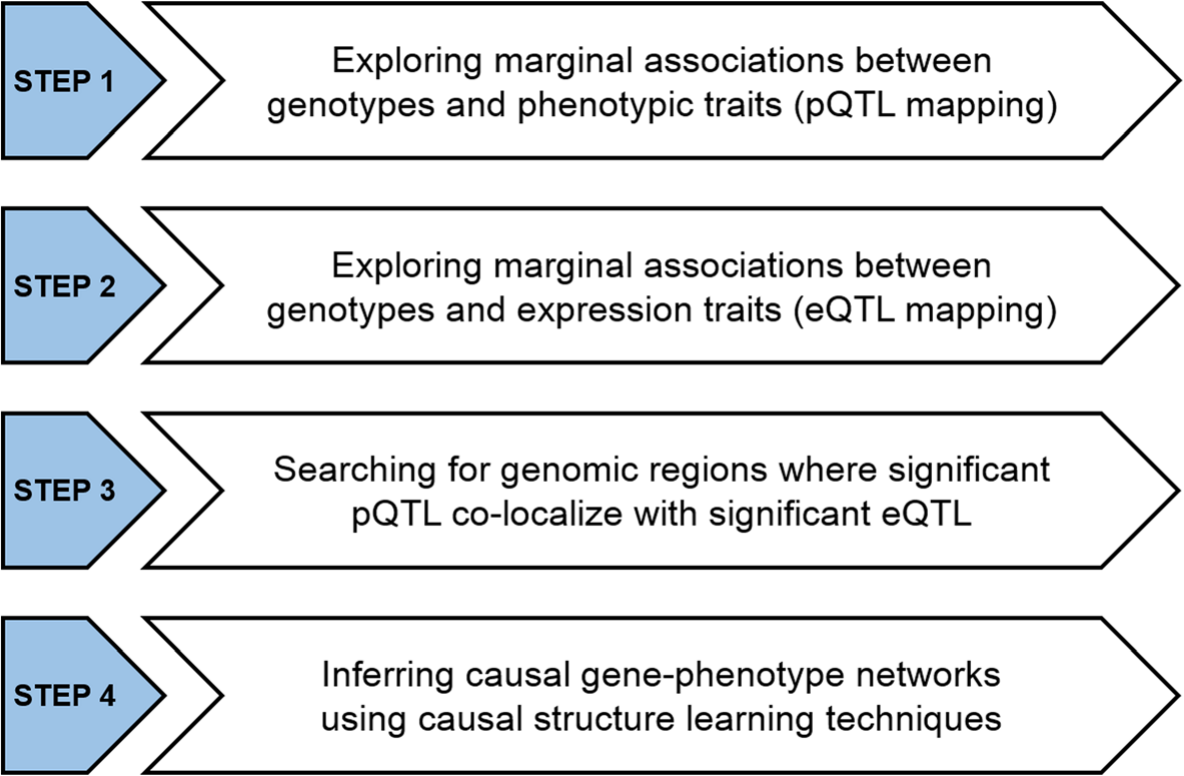
Multistep procedure for inferring causal gene-phenotype networks integrating phenotypic, genotypic, and transcriptomic data

Pig breeding programs have been mainly focused on the improvement of growth rate and production efficiency, such as average daily gain, food conversion ratio, dressing percentage, and lean meat content. This strategy has favorably improved carcass fat content, including backfat thickness but adversely affected intramuscular fat content, as well as some meat quality traits [15]. In this context, information regarding molecular networks underlying fat deposition and muscularity can be used to optimize management practices and selection strategies in pig breeding. As such, the main objective of this study was to assess gene-phenotype network reconstruction integrating phenotypic, genotypic, and transcriptomic data obtained from an F_2_ Duroc x Pietrain resource population. Causal networks were inferred using a multistep procedure (Fig. 1). Briefly, we firstly explored marginal associations between genotypes and phenotypic and expression traits through the use of whole-genome scans, and then, in those regions where several eQTL and pQTL co-localize, we attempted network reconstruction using causal structural learning algorithms (Fig. 1). As a proof of principle of the practical significance of this integrative approach, we show here the construction of causal molecular networks underlying carcass fat deposition and loin muscle weight.

## Methods

### Ethics statement

Experimental procedures were approved by the All University Committee on Animal Use and Care at Michigan State University (AUF# 09/03-114-00).

### Animals

Animals from a three-generation resource pig population developed at Michigan State University were used for this study. This population is an F_2_ cross originated from 4 F_0_ Duroc sires and 15 F_0_ Pietrain dams. The full pedigree consists in a single large family of 19 F_0_, 56 F_1_ (including 50 females and 6 males), and 954 F_2_ animals. Further details of population development and animal management are found in Edwards et al. [16, 17].

### Phenotypic data

Over 60 different phenotypes related to growth, body composition, carcass merit and meat quality were collected on the Michigan State University F_2_ Duroc x Pietrain resource population. In this study, we focused on carcass and meat quality phenotypes that were measured on or were directly related to longissimus dorsi (loin) muscle. Details of carcass and meat quality phenotype collection were published in Edwards et al. [17]. Briefly, carcass traits collected included loin muscle weight, and loin muscle pH and temperature at 45 min and 24 h postmortem. During carcass fabrication, measurements of loin muscle area and off-midline 10th-rib backfat thickness were also recorded. In addition, a section of the loin was further evaluated for meat quality traits. Traits included subjective and objective color, marbling and firmness. Samples were also evaluated for proximate composition, including moisture, intramuscular fat and protein. A trained sensory panel evaluated samples for juiciness, tenderness, connective tissue and off-flavor.

### Genotypic data

Animals from the Duroc x Pietrain resource population, including F_0_, F_1_, and F_2_ individuals, were genotyped for 124 dinucleotide microsatellites genetic markers (3-9 markers per chromosome) at a commercial laboratory (GeneSeek Inc., Lincoln, NE). This genotype information was used to derive breed of origin probabilities across the genome of F_2_ animals. In particular, probabilities of each F_2_ individual being homozygous for Duroc alleles (*P*_11_), homozygous for Pietrain alleles (*P*_22_), or heterozygous (*P*_12_ *or P*_21_) were estimated at each microsatellite marker and at 11 equidistant inter-marker positions, yielding in total 1,279 putative QTL positions spanning the whole pig genome. Breed of origin probabilities were derived assuming that the parental breeds (i.e., Duroc and Pietrain) were fixed for alternative QTL alleles [18].

### Transcriptomic data

Longissimus dorsi (loin) muscle tissue was sampled from a total of 176 F_2_ individuals during slaughter. The transcriptome of this tissue was measured for each of the 176 F_2_ animals using a pig whole-genome 70-mer oligonucleotide microarray. This microarray includes 20,400 annotated oligonucleotides spanning the whole swine genome. Details regarding tissue sample collection, sample preparation, microarray hybridization and pre-processing data were reported in Steibel et al. [19]. The resulting normalized gene expression data (intensity values) were expressed in the log2 scale.

### Genome-wide linkage analysis

The dataset for analysis included several phenotypes, genotype information, and gene expression data for a total of 171 F_2_ individuals. Two complementary whole-genome scans were performed: first, we carried out a classical phenotypic QTL mapping (pQTL) integrating phenotypic and genotypic data, and second we performed an expression QTL mapping (eQTL) integrating transcriptional profiling with genotypic data.

For the pQTL mapping, the following linear model was fitted separately to each phenotype directly related to loin muscle (e.g., loin muscle weight, loin muscle area):

$$ {y}_{ijk}=\mu +se{x}_i+ grou{p}_j+ carcw{t}_k\cdot \beta +{c}_k\cdot \alpha +{e}_{ijk} $$

where *y*_*ijk*_ is the phenotypic trait under study of the *k*^*th*^ F_2_ animal within the combination of *sex*_*i*_ and⋅ *group*_*j*_, ⋅ *μ* is the general mean, *sex*_*i*_ represents the fixed effect of the sex of the *k*^*th*^ animal, *group*_*j*_ represents the fixed effect of the slaughter group of the *k*^*th*^ animal, and *carcwt*_*k*_ is the carcass weight of the *k*^*th*^ animal as a linear covariate. As mentioned before, the additive QTL coefficient $c$ was derived assuming that the parental breeds were fixed for alternative alleles. In particular, *c*_*k*_ = *P*_11_ − *P*_22_ is the conditional expectation of the number of Duroc alleles carried by the *k*^*th*^ animal. The significance of the additive pQTL effect *α* at each of the 1,279 putative pQTL positions for each phenotypic trait was tested using an *F*-test by comparing the full model to the reduced model without the QTL effect. Significance thresholds of 5 % at genome-wise level were determined through the use of permutation tests [20].

For the eQTL mapping, the following linear mixed model was fit to normalized log-intensity data:

$$ {w}_{ijkl}=\mu + dy{e}_i+ arra{y}_j+\operatorname{se}{x}_k+{c}_l\cdot \alpha +{e}_{ijkl} $$

where *w*_*ijkl*_ is the normalized log-intensity for each oligonucleotide measured in the loin muscle of the *l*^*th*^ animal, *μ* is the general mean, *dye*_*i*_, *array*_*j*_, and *sex*_*k*_ are effects accounting for systematic variation in the microarray experiment of the *l*^*th*^animal; *dye* and *sex* were fitted as fixed effects, while *array* was fitted as a random effect. As described above, *c*_*l*_ is the additive QTL coefficient of the *l*^*th*^ animal calculated as *P*_11_ − *P*_22_. The significance of the additive eQTL effect *α* at each of the 1,279 putative eQTL positions and for each expression trait was tested using a likelihood ratio test by comparing the aforementioned model to a reduced model without QTL effect. The *p*-values were corrected for multiple testing across all expression traits and positions using Benjamini and Hochberg procedure [21].

### Causal structural learning

Causal structures are represented here using graphical models; these models combine the rigor of a probabilistic approach with the intuitive representation of relationships given by graphs. Graphical models are composed of two parts: a set ***V*** of random variables describing the quantities of interest, and a graph *G* = (**V**, *E*) in which each vertex *ν* ∈ ***V*** is called node, and each edge *e* ∈ *E*, also called arc or link, is used to express the dependence structure of the data, i.e., the set of dependence relationships among the variables in ***V*** [22].

There are several structure learning algorithms that can be used to infer the network structure underlying a given set of correlated variables, assuming that conditional independencies in the joint probability distribution of these variables mirror *d*-separations in the causal structure (for more details, see [6, 23]). One of such algorithms is the Inductive Causation (IC) algorithm, which is able to search for a class of minimal causal structures that are compatible with the conditional independencies implied by the joint distribution of the data [24]. The IC algorithm, when applied to a set ***V*** of variables, can be described as follows:

Step 1. For each pair of variables *A* and *B* in ***V***, search for set of variables ***S***_*AB*_ ⊂ ***V*** such that *A* and *B* are independent given **S**_*AB*_. If there is no such set, i.e., if *A* and *B* are dependent for every possible **S**_*AB*_, then place an undirected edge between *A* and *B*.

Step 2. For each pair of non-adjacent variables *A* and *B* with a common adjacent variable *C*, search for a possible set **S**_AB_ containing *C* such that *A* and *B* are independent given **S**_AB_. If there is no such set, then assign the direction of the edges A ‐ C and C ‐ B as *A* → *C* and *C* ← *B*.

Step 3. In the partially directed graph returned by the previous two steps, orient as many of the undirected edges as possible in such a way that it does not result in (i) new v-structures (i.e. new unshielded colliders) or (ii) directed cycles.

Even though the IC algorithm provides the theoretical framework for causal structural learning using conditional independent tests, its application to practical problems with several variables is hampered due to the exponential number of possible conditional independence relationships to be tested. This has led to the development of more efficient algorithms. Here, we have used one of such algorithms, the Incremental Association Markov Blanket (IAMB) algorithm [25]. The IAMB algorithm first learns the Markov Blanket of each variable in the dataset; the Markov Blanket of a given variable *Y* is defined as the minimal set of variables conditioned on which all other variables are probabilistically independent of the target *Y*. This preliminary step reduces the number and the size of the subsets considered in the conditional tests, and hence results in a lower computational complexity without compromising the accuracy of the resulting causal network [25].

Practical application of the IAMB algorithm involves performing a set of statistical decisions using conditional independence tests. In the context of normally distributed variables, these tests are functions of the partial correlation coefficients *ρ*_*XY*|**W**_ between *X* and *Y* given **W**. Here, we used the Fisher’s Z test, which involves a transformation of the linear correlation coefficient and is defined as:

$$ Z\left(X,Y\left|\mathbf{W}\right.\right)=\frac{1}{2}\cdot \sqrt{n-\left|\mathbf{W}\right|-3\cdot } \log \frac{1+{\widehat{\rho}}_{XY\left|\mathbf{W}\right.}}{1-{\widehat{\rho}}_{XY\left|\mathbf{W}\right.}} $$

which has an approximate normal distribution with mean zero and variance 1, i.e., *Z*(*X*, *Y*|**W**) ∼ *N*(0, 1). After the structure of the network was learned, the estimation of the parameters of the local distributions was performed using maximum likelihood. Since the variables under study are continuous, the causal parameters take the form of regression coefficients. Furthermore, the stability of the structure of the causal networks was evaluated using Jackknife resampling. By leaving out one observation per time from the dataset, we could evaluate the stability of each edge in the original network in terms of presence (binary variable; presence or absence in the resampled network) and direction (three possible outcomes; same direction as the original arrow, opposite direction, or undirected arc). All these analyses were performed using the bnlearn package [26] implemented in the R language/environment [27].

## Results

### pQTL and eQTL analysis

**Fig. 2:**
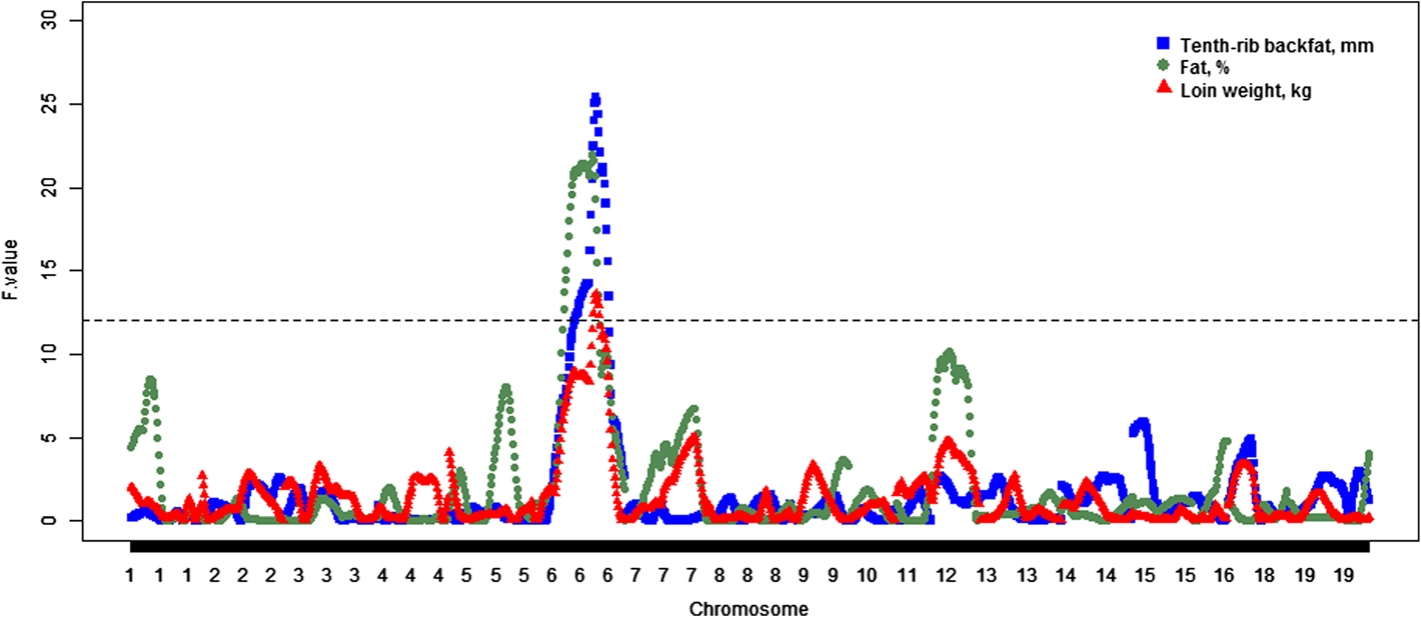
Genome scan results for loin muscle weight (*red*), off-midline 10th-rib backfat thickness (*blue*), and average intramuscular fat percentage (*green*). The horizontal line indicates the genome-wise significance level of 5 %

The first step in this study was to perform a classical whole-genome scan integrating phenotypes with genotypic information (pQTL mapping). We focused on carcass and meat quality traits that were measured on or were directly related to longissimus dorsi (loin) muscle. Three traits, namely loin muscle weight, off-midline 10th-rib backfat thickness (BF10), and average intramuscular fat percentage, showed significant pQTL at 5 % genome-wise significant level. Remarkably, these three significant pQTL mapped to the same genomic region on chromosome 6 (SSC6) of the pig genome (Fig. 2). It is worth noting that the Duroc allele was significantly associated with an increase in backfat thickness and intramuscular fat percentage, reducing at the same time the weight of the loin muscle. Overall, these findings provide some evidence of the existence of a genomic region on SCC6 with additive pleiotropic effects affecting both fat deposition and muscularity.

**Fig. 3:**
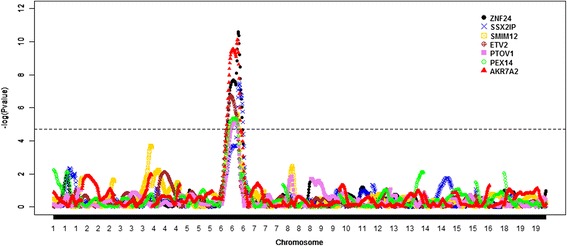
Genome scan results for seven expression traits that show significant eQTL on chromosome 6 (SSC6). The horizontal line indicates *P-*value = 1.9 × 10^−5^ (FDR ≤ 0.20). All these genes are located on SCC6 and hence these seven significant eQTL can be considered as local or cis-eQTL

The second step in this study was to perform another QTL mapping but now integrating transcriptional profiling with genotypic data (eQTL mapping). Here, we focused on the genomic region of SCC6 that was significantly associated with the three phenotypic traits discussed above. In this context, seven significant eQTL (FDR $\le$ 0.20) were detected in this region of the pig genome (Fig. 3). These seven eQTL were associated with the level of expression of the following seven genes: zinc finger protein 24 (*ZNF24*), aldo-keto reductase family 7, member A2 (*AKR7A2*), synovial sarcoma, X breakpoint 2 interacting protein (*SSX2IP*), ets variant 2 (*ETV2*), small integral membrane protein 12 (*SMIM12*), peroxisomal biogenesis factor 14 (*PEX14*), and prostate tumor overexpressed 1 (*PTOV1*). Genes *ZNF24*, *ETV2*, and *PEX14* were over-expressed in animals carrying the Duroc allele, while *AKR7A2*, *SSX2IP*, *SMIM12*, and *PTOV1* showed higher expression in animals with the Pietrain allele. It is important to note that all these genes are located on SCC6 and hence these seven significant eQTL can be considered as local or cis-eQTL.

### Causal networks

**Fig. 4:**
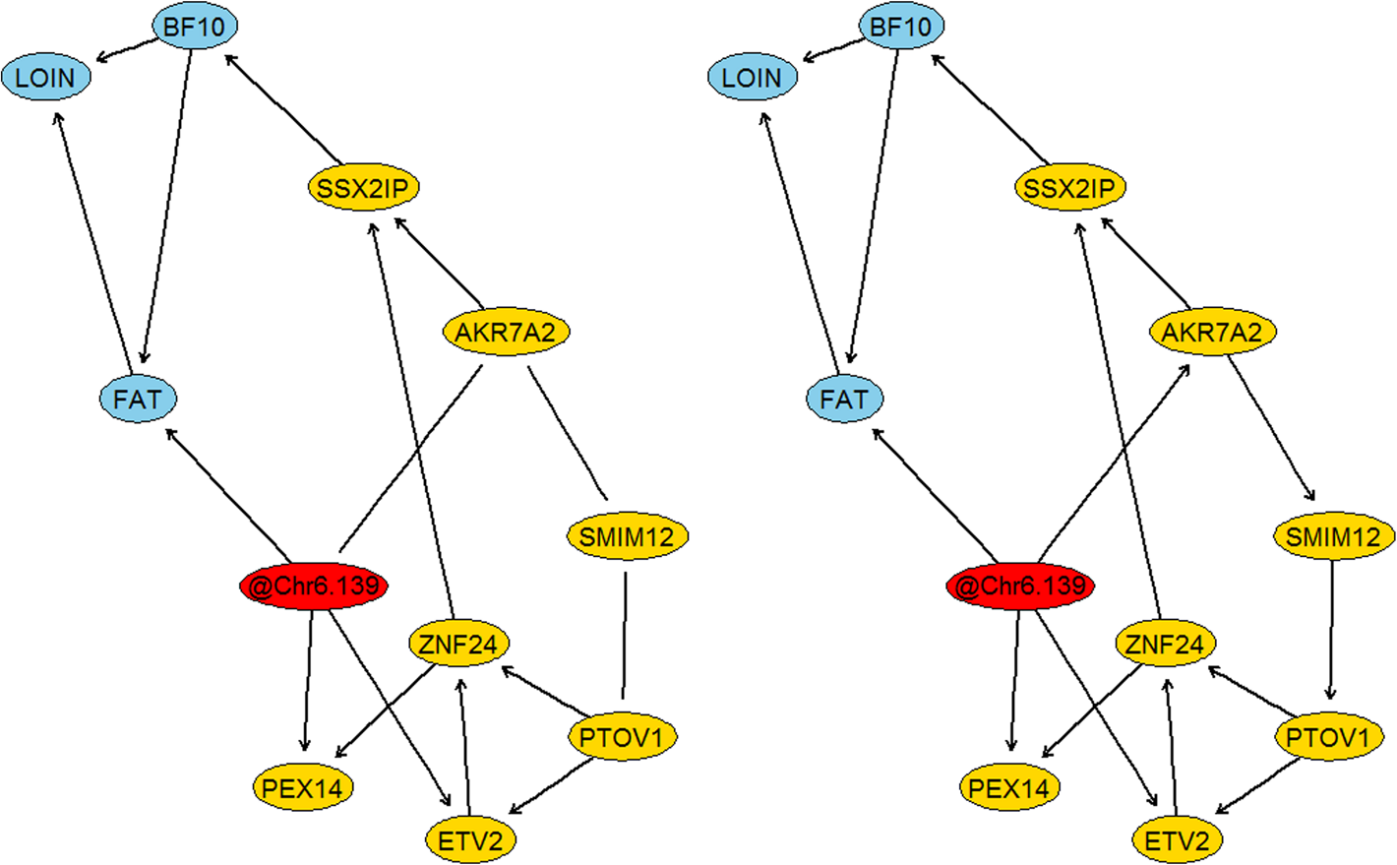
Causal networks integrating phenotypic (*blue*), genotypic (*red*) and transcriptomic (*yellow*) data. Left **a**: causal network inferred without using any prior information. Right **b**: causal network inferred after incorporation of @Chr6.139 $\to$ AKR7A2 as prior knowledge

The pQTL and eQTL analyses showed that there are at least three phenotypic traits and seven different gene expression traits significantly associated with the same genomic region on SCC6 of the pig genome. In order to decipher potential causal links among these variables, the IAMB algorithm (efficient constraint-based algorithm based on the inductive causation algorithm) in conjunction with Fisher’s Z test to assess for conditional independence (*α* = 0.05) were used to infer the functional relationships involving these 10 phenotypic and expression traits. In particular, the causal structural learning was performed using adjusted phenotypic and expression traits (i.e., corrected by systematic effects), and the most significant genetic marker located in this region. Interestingly, without using any prior information, the IAMB algorithm could reconstruct a partially directed acyclic graph with only three undirected edges (Fig. 4a). In this sense, the links between the genetic marker (labeled as @Chr6.139) and AKR7A2, between SMIM12 and AKR7A2, and between PTOV1 and SMIM12 remained unresolved (i.e. undirected). Now, using as prior knowledge that the undirected link between the genotype and AKR7A2 should be set as @Chr6.139⋅ → ⋅AKR7A2 (i.e., genotype may have a causal effect on the gene expression but not the opposite), then the algorithm could reconstruct a fully directed acyclic graph (Fig. 4b). Remarkably, based on the causal graphical model, the genetic marker is marginally associated (through direct and indirect paths) with all the other variables, i.e., phenotypic and expression traits. These findings completely agree with our previous pQTL and eQTL results. In addition, even though the genotype is directly linked to one phenotype (@Chr6.139 ⋅ → ⋅FAT), in general the graphical causal model indicated that the effects of the genotype on the phenotypes are mediated by the expression of several genes.

**Fig. 5:**
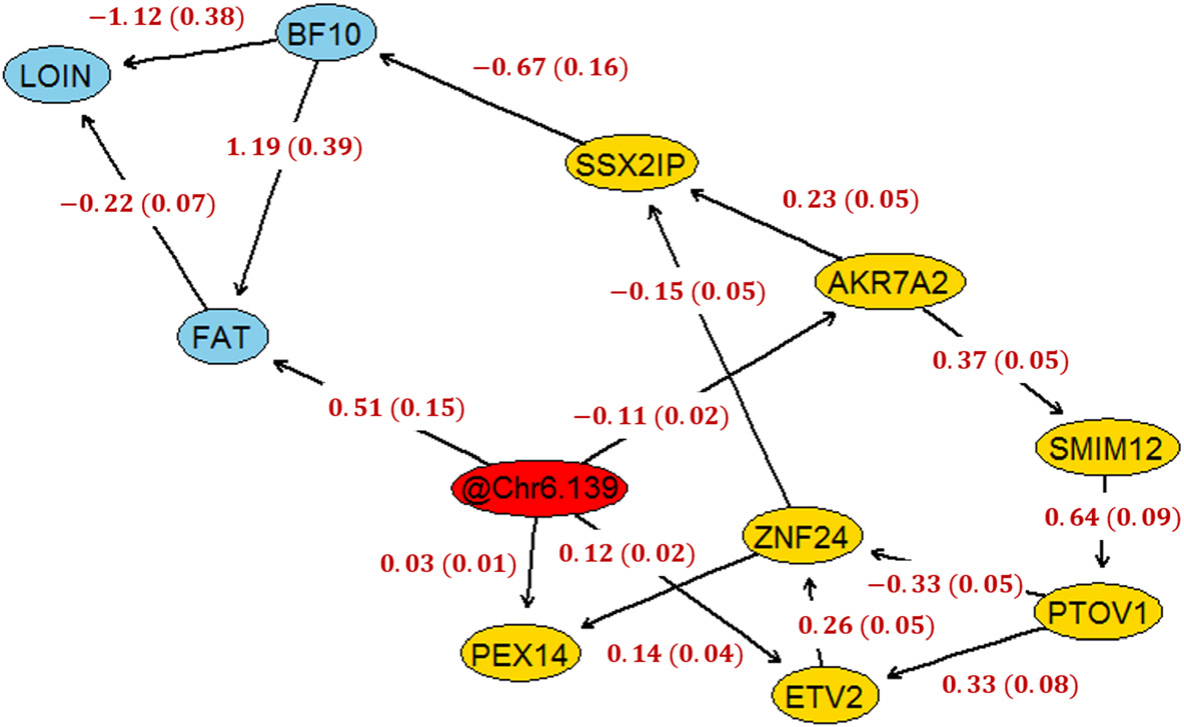
Maximum likelihood estimates for causal effects. Conditional on the inferred structure of the network, point estimates (*and standard errors*) of the causal parameter were estimated by Maximum Likelihood. The structure of the network was inferred integrating phenotypic (*blue*), genotypic (*red*) and transcriptomic (*yellow*) data

Conditionally on the structure of the network, point estimates of the causal parameters were estimated using maximum likelihood (Fig. 5). The genotype (Duroc allele) had a positive total effect on fat deposition (BF10 and FAT) and a negative total effect on loin muscle weight. These effects are in general mediated by the expression of several genes. In fact, there were in total 3 and 4 paths from @Chr6.139 to BF10 and FAT, respectively. All these paths showed a positive effect of the genotype on the phenotypes. In addition, there were in total 7 different paths from the genetic marker to LOIN; all these paths showed a negative effect of the genotype on loin muscle weight.

**Fig. 6:**
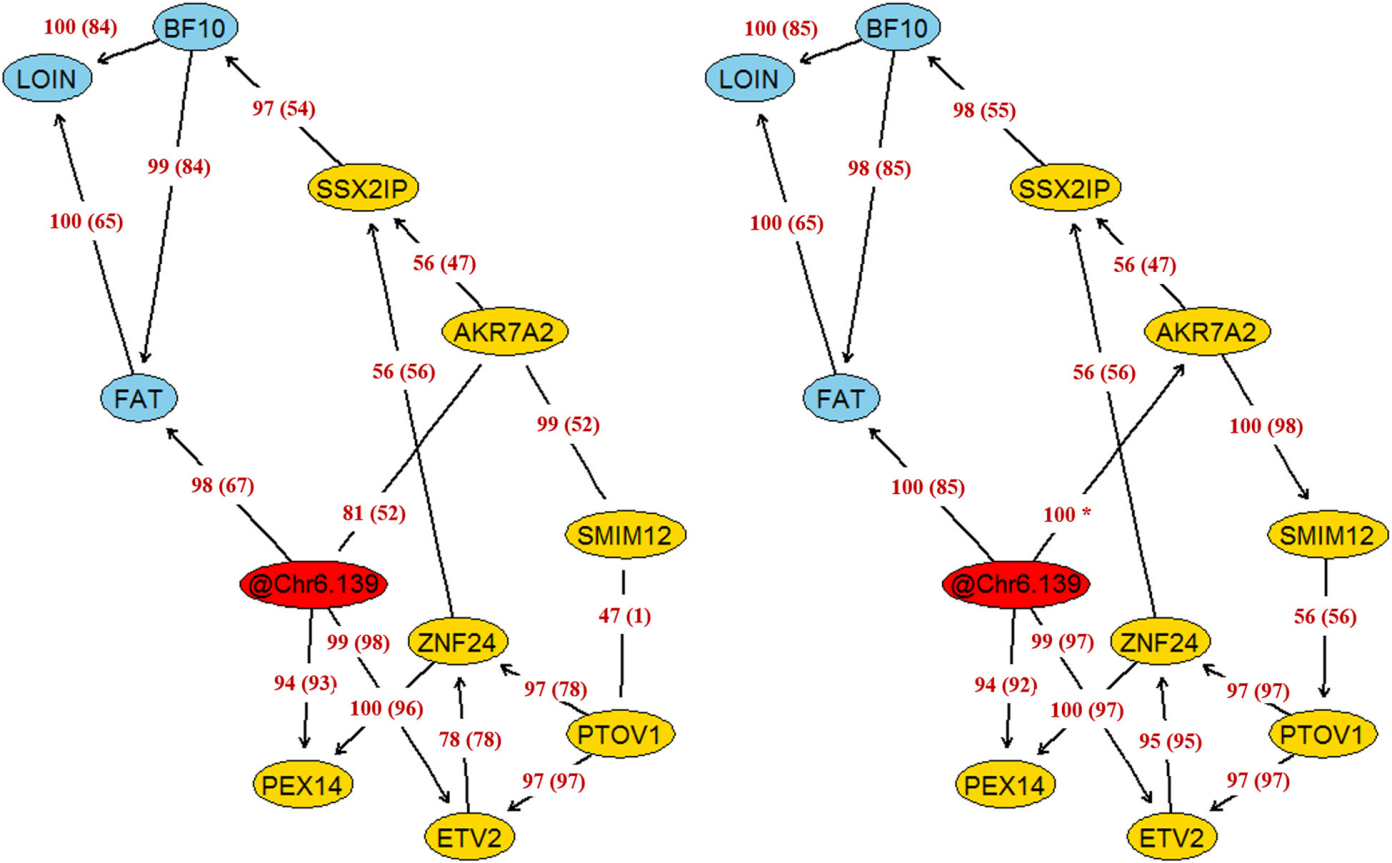
Evaluation of the stability of the network using Jackknife resampling. Results are expressed as frequency (*percentage*) that a given arc was presented (*with the same direction*) in the resampled networks. The structure of the networks was inferred integrating phenotypic (*blue*), genotypic (*red*) and transcriptomic (*yellow*) data

The stability of the network was evaluated using Jackknife resampling. In each iteration, the network was inferred from a new dataset which was created by removing one animal at a time from the original dataset. The structure of this new network was then compared with the original structure. In particular, we evaluated the stability of each link (presence or absence) and also the stability of the direction of the link. Figure 6 displays the results of the Jackknife resampling. There were no important differences in the stability of the links between networks constructed with or without prior information (setting or not the link @Chr6.139 ⋅ → ⋅AKR7A2 as known). Notably, the majority of the links and directions showed great stability. In fact, the arrows between phenotypic traits, and the links from the genetic marker to the intermediate variables remained in general unchanged. There were very few connections that were unstable (e.g., between SMIM12 and PTOV1), i.e., the removal of a single data point caused the absence of connection between the variables.

## Discussion

In this study, we have evaluated gene-phenotype network reconstruction integrating phenotypic, genotypic, and transcriptomic data obtained from a genetical genomics study performed in pigs. The dataset for analysis included carcass and meat quality phenotypes, genotypic information spanning the whole genome, and gene expression data measured in the longissimus dorsi (loin) muscle for a total of 171 F_2_ Duroc x Pietrain pigs. We focused on carcass and meat quality traits that were measured on or were directly related to loin muscle. The multistep procedure used for network reconstruction can be summarized as follows (see Fig. 1): first, we performed a classical QTL mapping for phenotypic traits (pQTL); second, we performed a new QTL mapping but now using the gene expression as a response variable (eQTL); third, we searched for genomic regions in the pig genome where significant pQTL co-mapped with several significant eQTL; and finally, using the information provided by these regions, we assessed gene-phenotype network reconstruction using causal structure learning techniques.

One genomic region on SSC6 showed remarkable results of particular interest for this study. In fact, controlling genome-wise significant level at 5 %, three relevant phenotypes, namely loin weight, off-midline 10th-rib backfat thickness, and average intramuscular fat percentage, showed significant QTL in this genomic region. In addition, seven significant eQTL (FDR⋅ ≤ ⋅0.20) were also detected in this particular region of the pig genome. Many of these genes have important roles in cell proliferation and differentiation. It is worth noting that previous studies in pigs, including previous analyses of this same Michigan State University F_2_ Duroc x Pietrain resource population, have already reported significant QTL for fat deposition (e.g., 10th-rib backfat thickness, last lumbar vertebra backfat thickness, intramuscular fat, and marbling) and muscularity (e.g., ham weight, loin weight, and loin muscle area) in this region of SCC6 [17, 28–30]. Our findings showed that the Pietrain allele is negatively associated with fat deposition and positively associated with loin weight. These results support previous studies that found that Pietrain pigs have less backfat and larger longissimus dorsi muscle area compared to Duroc pigs [31, 32]. Overall, these two complementary whole-genome scans revealed an interesting genomic region on SCC6 with pleiotropic additive effects on fat deposition and muscularity, which is also significantly associated with the expression of several genes.

We further explored this genomic region using structural learning techniques in order to decipher potential causal relationships between phenotypic and expression traits. Remarkably, the output of the structural learning algorithm reflected all those marginal associations detected in the whole-genome scans. More importantly, the causal network showed that the effect of the genotype on the phenotypic traits is mainly mediated by the expression of several genes. In addition, our findings revealed that both fat deposition traits, off-midline 10th-rib backfat thickness and average intramuscular fat percentage, had a negative effect on loin muscle weight. Previous studies have reported that selection of pigs for less backfat thickness resulted in improved carcass lean meat content and loin muscle size, and also less intramuscular fat [15, 33]. Hence, our findings provide a causal explanation for this phenomenon.

Arguably one of the most relevant genes in the network is *ZNF24*, whose expression mediates the effects of the genotype on the phenotypes. *ZNF24* encodes a member of the family of Krüppel-like zinc finger transcription factors and has critical roles in cell proliferation and differentiation [34]. In our study, *ZNF24* showed higher expression in animals carrying the Duroc allele. Of particular interest, a recent study reported higher expression of *ZNF24* in loin muscle of Basque compared with Large White pigs [35]. Similarly to Duroc, the Basque breed shows high fat contents and high meat quality characteristics, and therefore, our findings provide further evidence of the potential association between *ZNF24* and fat deposition and meat quality merit in pigs. Another relevant gene is *SSX2IP*, which is located in the network just upstream of the phenotypic traits. *SSX2IP* showed a negative causal effect on backfat thickness, and was unsurprisingly overexpressed in animals carrying the Pietrain allele. *SSX2IP* has been shown to play a role in cell adhesion, actin cytoskeleton organization, and regulation of cell motility [36]. Our findings support this gene as a promising candidate for carcass lean meat content.

Knowledge about gene-phenotype networks can be used to predict the behavior of complex systems. For instance, in our study, the network model predicts that modulation of *ZNF24* expression level should lead to changes in the expression of *SSX2IP*. Recently, Li et al. [34] evaluated potential *ZNF24* target genes. For this purpose, the authors transiently overexpressed and silenced *ZNF24* and then applied microarray assay in order to identify target genes. Notably, the overexpression of *ZNF24* significantly decreased the expression of *SSX2IP*, as predicted by our network. In addition, the silencing of ZNF24 resulted in a significant overexpression of *SSX2IP* [34]. Therefore, these results support the causal relations inferred in our study.

Overall, we have detailed a multistep procedure for inferring causal networks integrating phenotypic, genotypic, and transcriptomic data. We have applied this procedure for deciphering gene-phenotype networks underlying fat deposition and muscularity in pigs. Our findings shed light on the antagonist relationship that exists between carcass fat deposition and lean meat content. More generally, the procedure described here can be easily applied to unravel causal molecular networks underlying complex phenotypes in livestock species.

## Availability of supporting data

The gene expression data were deposited in the NCBI Gene Expression Omnibus database (http://www.ncbi.nlm.nih.gov/geo/) [accession number GSE23351].
